# Using the Six-Minute Walk Test to Evaluate Functional Capacity of Children Undergoing a Surgical Repair of Congenital Heart Disease: Two Case Reports

**DOI:** 10.7759/cureus.78017

**Published:** 2025-01-26

**Authors:** Ritik V Daf, Lajwanti Lalwani

**Affiliations:** 1 Cardiorespiratory Physiotherapy, Ravi Nair Physiotherapy College, Datta Meghe Institute of Higher Education and Research, Wardha, IND

**Keywords:** 6-minute walk test, congenital heart disease (chd), exercise capacity, pediatrics, valve surgery

## Abstract

Congenital heart disease (CHD) presents a range of structural abnormalities in the heart that are present at birth. Advances in surgical techniques have significantly improved outcomes for children with CHD. Common surgical procedures include repair of septal defects, such as atrial septal defects (ASDs) and ventricular septal defects (VSDs), as well as correction of complex anomalies such as tetralogy of Fallot (TOF) and transposition of the great arteries. Despite these advancements, it is crucial to assess the exercise capacity of children with CHD. This evaluation provides insights into their cardiovascular function and helps tailor appropriate exercise recommendations. Children with CHD often exhibit reduced exercise tolerance due to factors such as altered heart function, limited blood flow, or impaired oxygen delivery. Assessing their exercise capacity through standardized tests, such as the six-minute walk test (6MWT) or cardiopulmonary exercise test, helps clinicians gauge their functional abilities and determine any limitations. Understanding a child's exercise capacity guides medical management and also aids in designing personalized exercise programs to promote cardiovascular health and overall well-being. Regular assessments can track changes over time, ensuring optimal care and enhancing the quality of life (QOL) for children living with CHD. These two case reports examine the exercise capacity of two children with CHD who underwent surgery for VSD. Both children participated in the 6MWT, covering 223 and 183 meters, respectively. The physiological responses of these two CHD patients during the exercise test are discussed in this case series. This case series provides information regarding the cardiovascular adjustment during the 6MWT and various causes that affected them to complete the 6MWT.

## Introduction

Congenital heart diseases (CHD) represent a spectrum of structural abnormalities in the heart that are present at birth, affecting approximately 1% of newborns worldwide. These defects can involve the heart walls, valves, or blood vessels, leading to disruptions in its normal function [1]. There are various types of CHD, each with distinct characteristics and impact on cardiovascular health. The CHD is classified as left to right shunt or right to left shunt. Common types include ventricular septal defects (VSD), atrial septal defects (ASD), and aortic valve stenosis [2]. Treatment of choice for CHD is mainly corrective surgery or palliative surgery. Core surgeries for CHD focus on correcting these structural abnormalities to restore proper heart function and prevent long-term complications. Among these procedures, closure of VSD and ASD with repair of valve defects, stand as pivotal interventions. These surgeries are vital in alleviating symptoms such as cyanosis, heart failure, and impaired growth [3]. The inflammatory response and tissue trauma following surgery also contribute to temporary muscle weakness and fatigue.

Additionally, postoperative pain and medication side effects may limit mobility and hinder exercise tolerance. This decline in exercise capacity underscores the importance of structured rehabilitation programs aimed at improving cardiovascular fitness, muscle strength, and overall functional capacity. Post-surgery assessment is crucial, especially in pediatric patients [4,5]. Evaluation often involves sub-maximal tests such as the six-minute walk test (6MWT), which measures exercise capacity and functional status. Parameters such as oxygen saturation, heart rate, blood pressure, and perceived exertion on the Borg rating of perceived exertion (RPE) scale offer valuable insights into recovery progress. The 6MWT is a non-invasive test frequently used to measure exercise capacity in post-cardiac surgery patients [6]. During the 6MWT, patients are tasked with walking as far as possible within a 20-meter straight path, turning at each end. The goal is to cover the maximum distance in the allotted time of 6 minutes. Patients are encouraged to move at their own pace without direct influence from the examiner, emphasizing a natural and self-selected speed throughout the test [7].

The assessment of the 6MWT in the pediatric population following cardiac surgeries lacks sufficient evidence. By understanding their postoperative functional status through the 6MWT, healthcare providers can design interventions to enhance cardiovascular fitness and muscle strength. This approach not only aids in immediate recovery but also lays the foundation for lifelong habits of physical activity, promoting an active and healthy lifestyle into adulthood. Further research and utilization of the 6MWT in this context can lead to improved outcomes and better long-term health prospects for pediatric cardiac surgery patients. Here, we present the case of two patients' exercise capacity assessment with the help of a 6MWT.

## Case presentation

Case 1

**Figure 1:**
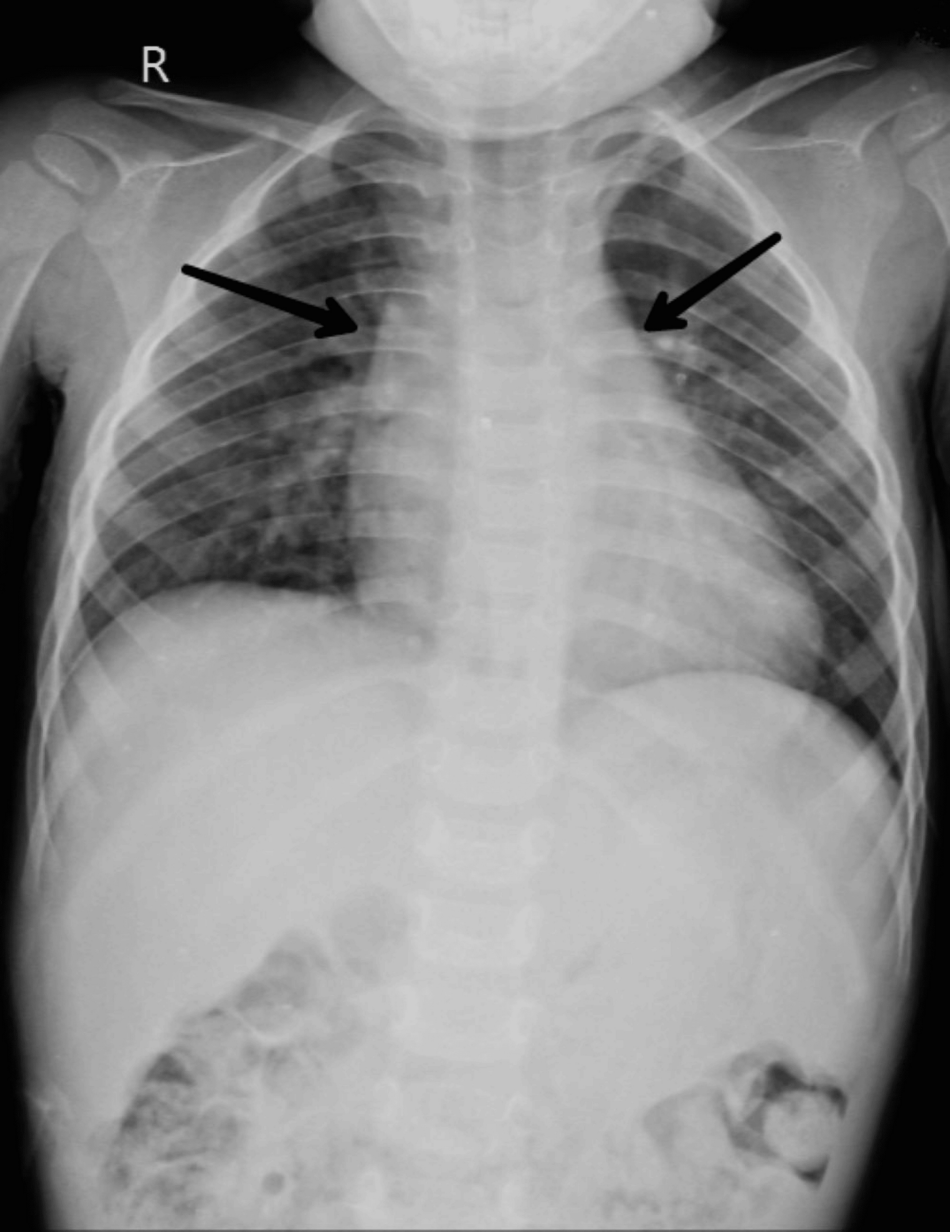
Preoperative AP chest radiograph AP: Anteroposterior

A 3-year-old male child, weighing 9 kg, presented with recurrent cough and cold. Transthoracic echocardiography (TTE) revealed CHD, moderate size perimembranous VSD restricted by stereo lithography aneurysm, small patent ductus arteriosus (PDA), and good left ventricular function and sinus rhythm. The serial x-ray of the patient showed mediastinum widening, cardiomegaly, and increased pulmonary vasculature (Figure 1).

The patient was admitted to the cardiovascular thoracic surgery (CVTS) ward and operated on for VSD closure; a PDA ligation synthetic fabric dacron (SFD) patch was used. After the surgery, the patient was moved to the CVTS intensive care unit (ICU). On postoperative day 7, the patient was moved to the CVTS ward. The patient was discharged on postoperative day 8. On discharge day, a cardiorespiratory assessment was taken using 6MWT. During the test, saturation levels did not drop. Table 1 and Table 2 show the patient's pre- and post-assessment findings.

**Table 1:** Physiological findings of case 1 RPE: Rating of perceived exertion Borg RPE scale: 6 - No exertion at all 9 - Very light exertion 13 - Somewhat hard exertion 15 - Hard exertion 20  - Maximum exertion

Assessment	Pre-test Results	Post-test Results	Normal Ranges
Peripheral capillary oxygen saturation	96%	96%	92% to 100%
Respiratory rate	22 breaths/min	30 breaths/min	22 to 34 breaths/min
Pulse rate	94 beats/min	126 beats/min	80 to 140 beats/min
Blood pressure	90/70 mmHg	95/75 mmHg	89-112/46-72 mmHg
Borg RPE scale	6	12	-

**Table 2:** Findings of case 1

Assessment	Results
Distance covered	223 meters
Breaks	3 minutes 20 seconds (30 seconds)
Challenges faced by therapist	Momentarily distracted by the sight of a family member

Case 2

**Figure 2:**
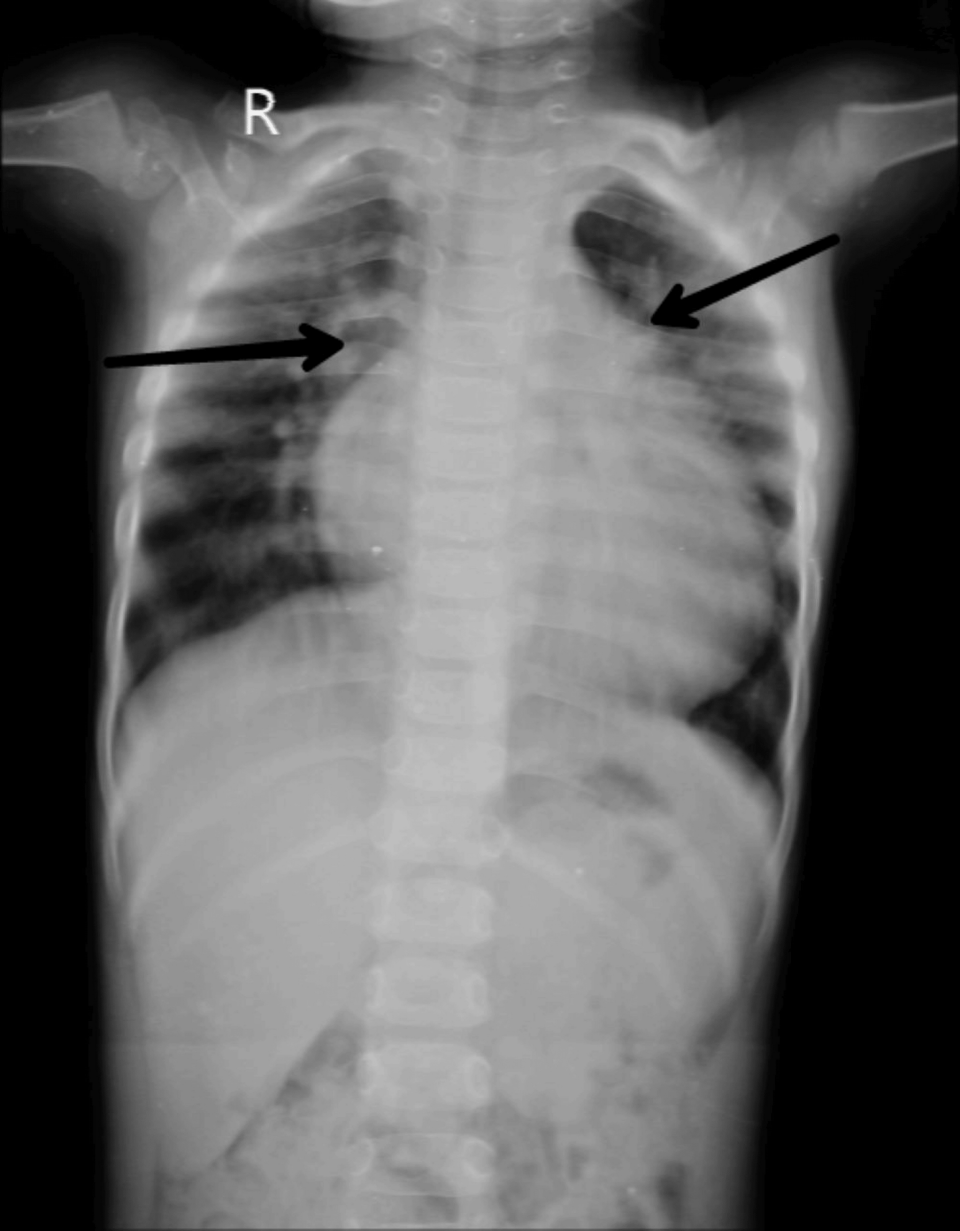
Preoperative AP chest radiograph AP: Antroposterior

A 3-year-old male child, weighing 10 kg, presented with recurrent cough and cold. TTE revealed CHD, small subaortic VSD, right coronary cusp prolapsed, and mild aortic regurgitation and sinus rhythm. The serial x-ray of the patient showed mediastinum widening, cardiomegaly, and increased pulmonary vasculature (Figure 2).

The patient was admitted to the CVTS ward and operated on for VSD closure; an SFD patch was used. After the surgery, the patient was moved to the CVTS ICU and monitored. On postoperative day 6, the patient was moved to the CVTS ward. The patient was discharged on postoperative day 9. On discharge day, a cardiorespiratory assessment was taken using 6MWT. During the test, saturation levels did not drop. Table 3 and Table 4 show the patient's pre- and post-assessment findings.

**Table 3:** Physiological findings of case 2 RPE: Rating of perceived exertion Borg RPE scale: 6 - No exertion at all 9 - Very light exertion 13 - Somewhat hard exertion 15 - Hard exertion 20 - Maximum exertion

Assessment	Pre-test Results	Post-test Results	Normal Ranges
Peripheral capillary oxygen saturation	96%	98%	92% to 100%
Respiratory rate	21 breaths/min	28 breaths/min	22 to 34 breaths/min
Pulse rate	114 beats/min	136 beats/min	80 to 140 beats/min
Blood pressure	102/76 mmHg	110/80 mmHg	89-112/46-72 mmHg
Borg RPE scale	8	13	-

**Table 4:** Findings of case 2

Assessment	Results
Distance covered	183 meters
Breaks	4 minute 28 seconds (discontinued later)
Challenges faced by therapist	Began weeping during the test

## Discussion

Patients typically get discharged from the hospital around 4 to 7 days after post-cardiac surgeries, depending on the procedure's complexity, individual health status, and recovery progress. Advances in surgical techniques and postoperative care have contributed to shorter hospital stays, promoting faster recoveries while ensuring patient safety and well-being [8,9]. It has been reported that physical capacity in children with CHD is lower compared to healthy controls with limited exercise capacity and a shorter lifespan related to health [10]. Limited exercise capacity favors a more sedentary lifestyle, a situation that can be maintained into adulthood [11]. Exercise capacity is one of the main factors when assessing health-related quality of life (QOL), prognosis, risk of morbidity, and early mortality from cardiovascular, metabolic, or respiratory disease [12]. It can be evaluated by a standardized laboratory test such as the cardiopulmonary exercise test or standardized field tests such as 6MWT, shuttle walking test, time up and go, or similar tests [13].

Assessing the exercise capacity of children and adolescents with CHD is crucial for enhancing their functional status and overall QOL. This evaluation plays a pivotal role in identifying potential poor prognostic factors associated with increased risks of morbidity and mortality.

Geiger et al. found that the average distance traveled by healthy individuals in the 3- to 5-year age group was 544 meters. In our assessment, our patients covered 183 and 223 meters, respectively. This comparison underscores the impact of CHD and postoperative recovery on exercise capacity in pediatric patients [14]. The assessment of 6MWT in the pediatric population following cardiac surgeries lacks sufficient evidence. However, it is crucial to evaluate the exercise capacity of these patients to develop a personalized exercise regimen for their rehabilitation. Enhancing exercise capacity can support patients in maintaining an active lifestyle into adulthood.

In assessing 6MWT in children, we encountered challenges during the test, with one child being distracted and pausing mid-way while another started weeping midway. These interruptions highlight the need for adjustments in the 6MWT protocol when working with pediatric patients. These modifications aim to create a more engaging and supportive testing environment, addressing issues, such as attention span and emotional comfort. Overcoming these challenges requires tailored adjustments to enhance engagement and comfort during the test, fostering a more cooperative environment. Improving communication strategies, incorporating interactive elements, and considering child-friendly incentives can aid in achieving more reliable and insightful results from 6MWT in pediatric patients. These modifications are crucial for the effective evaluation of exercise tolerance and functional capacity in this young age group. Patient discharge assessment post-cardiac surgery is essential for ensuring optimal recovery and reducing readmission rates. Effective assessment helps healthcare providers tailor postoperative care plans, including medication management, lifestyle modifications, and follow-up appointments, promoting continuity of care and preventing complications [15].

## Conclusions

The presented case reports highlight the response of 6MWT in pediatric CHD patients operated for VSD. The 6MWT emerges as a valuable tool for evaluating exercise capacity and functional outcomes post-surgery. The 6MWT can be effectively utilized in cooperative patients, particularly with pediatric populations. However, limitations arise when children may not readily obey commands, so modifications are necessary for accurate assessments. Prior evaluations on cooperation levels and establishing a relationship with the child can significantly impact the test's reliability and outcomes. Further studies are needed to incorporate additional components, enhancing cooperation in pediatric patients during 6MWT.
